# Retraction: Blocking TRPV1 in Nucleus Accumbens Inhibits Persistent Morphine Conditioned Place Preference Expression in Rats

**DOI:** 10.1371/journal.pone.0230396

**Published:** 2020-03-06

**Authors:** The PLOS ONE Editors

After this article [1] was published, concerns were raised about results reported in Figs 2 and 3:

- There appear to be several vertical and horizontal discontinuities in both panels of Fig 2A.
- The 1w panel of Fig 3A appears similar to the 3w panel of Fig 3B, except for the dark circular features representative of immunogold labelling.

In regard to Fig 2A, the authors provided raw image data and clarified that several bands had been rearranged in preparing the figure. The 1 mg/kg, 3 mg/kg, and 10 mg/kg morphine data were obtained using different blots, the control data were mislabeled in the figure, and the control lane in the original figure applied only to the 1 mg/kg samples. In addition, Fig 2A and 2B did not report data for saline-treated animals and as such were missing control findings needed to interpret the results.

For Fig 3, the authors provided a replacement image for the 3w panel of Fig 3B along with raw image data to support Fig 3A and the 1d, 1w panels of Fig 3B. They noted that the other electron microscopy data are no longer available, and they were unable to clarify the concern about the original figure.

In light of the unresolved concerns about Fig 3 and questions around the controls for the experiments reported in Fig 2, the *PLOS ONE* Editors retract this article.

G-DG notified the journal that all authors agree to retraction. The other authors either did not respond directly or could not be reached.
